# Biomass Allocation of Stoloniferous and Rhizomatous Plant in Response to Resource Availability: A Phylogenetic Meta-Analysis

**DOI:** 10.3389/fpls.2016.00603

**Published:** 2016-05-04

**Authors:** Xiu-Fang Xie, Yu-Kun Hu, Xu Pan, Feng-Hong Liu, Yao-Bin Song, Ming Dong

**Affiliations:** ^1^Key Laboratory of Hangzhou City for Ecosystem Protection and Restoration, College of Life and Environmental Sciences, Hangzhou Normal UniversityHangzhou, China; ^2^National Science Library, Chinese Academy of SciencesBeijing, China; ^3^State Key Laboratory of Vegetation and Environmental Change, Institute of Botany, Chinese Academy of SciencesBeijing, China

**Keywords:** biomass allocation, clonal reproduction, ontogenetic drift, optimal allocation theory, phylogenetic meta-analysis, trade-off, sexual reproduction, vegetative growth

## Abstract

Resource allocation to different functions is central in life-history theory. Plasticity of functional traits allows clonal plants to regulate their resource allocation to meet changing environments. In this study, biomass allocation traits of clonal plants were categorized into absolute biomass for vegetative growth vs. for reproduction, and their relative ratios based on a data set including 115 species and derived from 139 published literatures. We examined general pattern of biomass allocation of clonal plants in response to availabilities of resource (e.g., light, nutrients, and water) using phylogenetic meta-analysis. We also tested whether the pattern differed among clonal organ types (stolon vs. rhizome). Overall, we found that stoloniferous plants were more sensitive to light intensity than rhizomatous plants, preferentially allocating biomass to vegetative growth, aboveground part and clonal reproduction under shaded conditions. Under nutrient- and water-poor condition, rhizomatous plants were constrained more by ontogeny than by resource availability, preferentially allocating biomass to belowground part. Biomass allocation between belowground and aboveground part of clonal plants generally supported the optimal allocation theory. No general pattern of trade-off was found between growth and reproduction, and neither between sexual and clonal reproduction. Using phylogenetic meta-analysis can avoid possible confounding effects of phylogeny on the results. Our results shown the optimal allocation theory explained a general trend, which the clonal plants are able to plastically regulate their biomass allocation, to cope with changing resource availability, at least in stoloniferous and rhizomatous plants.

## Introduction

How plants allocate limiting resources among different life functions, e.g., growth vs. reproduction, survival vs. future growth, in response to the variation in their environments has been a central question in plant ecology for half a century. Bloom et al. (1985) vividly drew a parallel between a plant and a business firm, and articulated that plants like businesses must engage in long-term (i.e., reproduction) as well as short-term (i.e., growth) planning on resource allocation. The theory of allocation, borrowed from microeconomics, was firstly introduced to biology by Levins and MacArthur (cited in Cody, 1966) to describe the resource partitioning mode of iteroparous organisms and extended to the study of plants (Harper and Ogden, 1970). Subsequently, optimal allocation theory was proposed and suggested that plants regulate allocation of resources to their organs in response to variation in the environment in order to optimize the capture of resources (e.g., nutrients, light and water) essential for survival, growth and reproduction in a manner that maximizes fitness in changing environments (Bloom et al., 1985; McConnaughay and Coleman, 1999). According to the optimal allocation theory, plants should allocate resources to increase their uptake of the resource that is most limiting growth. It has been widely tested in different species (Bloom et al., 1985; Robinson, 1986; Johnson and Thornley, 1987; Levin et al., 1989; Hilbert, 1990; McConnaughay and Coleman, 1999). For example, plants allocated more biomass to leaf under low light intensity (Shipley and Meziane, 2002), and more biomass to root under low soil nutrients or water (Ericsson, 1995; Mony et al., 2007; Gonzáles et al., 2008). However, the optimal allocation theory has also been questioned for ignoring “ontogenetic drift,” which described the phenomenon of a trait changing in a predictable way as a function of plant growth or development (Evans, 1972; McConnaughay and Coleman, 1999). For instance, Coleman et al. (1994) found that plant biomass allocation was size-dependent and supported by the subsequent studies (Pino et al., 2002; Ogawa, 2003; Weiner, 2004; Huang et al., 2009).

The optimal allocation theory means that plastic resource allocation patterns result from environmental changes and are size-independent (Bloom et al., 1985). In this view, allocation is considered as a proportional process: “partitioning,” as in a pie chart, and usually analyzed as ratios (e.g., root mass: shoot mass, reproductive mass: vegetative mass) or fractions of total biomass (e.g., root mass: total mass, reproductive mass: total mass; Poorter and Nagel, 2000; Weiner, 2004). While the “ontogenetic drift” means that variable resource allocation patterns are genetically determined and size-dependent (Coleman et al., 1994; McConnaughay and Coleman, 1999). In this perspective, plant growth is allometric (allocation changing with size), and is generally showed by allometric analyses (Müller et al., 2000). However, we still do not know which theory can explain the general patterns of multiple species with their phylogenetic relations.

Around the above theories, there have been numerous studies focusing on resource allocation strategy of clonal plants, such as biomass allocation between growth and reproduction (Delph et al., 1993; Salonen, 1994; Li et al., 2001a; Van Zandt et al., 2003), between sexual and clonal reproduction (Hartnett, 1990; Cheplick, 1995; van Kleunen et al., 2002; Thompson and Eckert, 2004; Van Drunen and Dorken, 2012; Wang et al., 2013), between aboveground and belowground (Aerts et al., 1991; Cao and Ohkubo, 1998; King et al., 1999; Yang et al., 2009). Since clonal plants are mostly perennial and possess two modes of regeneration (Barrett, 2015), namely sexual reproduction by seed and clonal reproduction through a form of clonal growth (Richards et al., 2004), it is more complicated to understand resources allocation strategy of clonal plants. Resource allocation among vegetative growth, clonal reproduction and sexual reproduction may be age-related and not necessarily mutually exclusive in life history (Cheplick, 1995). Furthermore, higher plasticity of clonal plants allows them to modify the growth and development in response to changes in environmental conditions (Strand and Weisner, 2004). So, are there any general patterns of resource allocation of clonal plants in response to variation in availability of resources? If so, do these patterns depend on ontogeny, or the environment experienced by the clonal plants? Although, numbers of previous reviews have focused on various aspects of resource allocation (Bloom et al., 1985; Lovett Doust, 1989; de Kroon and Schieving, 1990; Reekie, 1999; Weiner, 2004), there has been no consistent conclusion drawn from quantitative analysis yet. The accumulation of studies in this topic, along with the development of meta-analysis, offers us an opportunity to examine the general trends of biomass allocation of clonal plant in response to changing resource availability.

Most studies have looked at allocation of biomass, as it generally reflects other resource available to an individual (Reekie and Bazzaz, 1987). In this study, we considered ramet biomass as a measure of resource allocated to vegetative growth, and further divided into two parts: aboveground part (shoot, leaf, and stem) and belowground part (root). Because some studies found that stolons and rhizomes have partly different functions (Dong and de Kroon, 1994) and because rhizomatous plants are less plastic than stoloniferous plants in response to changes in resource availability (Dong and de Kroon, 1994; de Kroon and Hutchings, 1995; Xie et al., 2014), we considered that biomass allocated to reproduction consisted of two parts: biomass allocated to clonal reproduction (rhizomes or stolons) and biomass allocated to sexual reproduction (flowers, seeds, and fruits; Table 1). Additionally, we analyzed the allocation patterns from two perspectives: absolute and relative biomass (Reekie and Bazzaz, 1987). The absolute biomass allocation to a component or activity (e.g., reproductive biomass) was a measure of the total quantity of the component or activity and was in relation to plant size, while the relative biomass allocation to that (e.g., reproductive biomass: total biomass) was a measure of the proportion of biomass devoted to it and was size-independent (Bazzaz et al., 2000). To take the evolutionary relationships of the multiple species involved into account, we adopted phylogenetic meta-analysis (PMA), an emerging method incorporating phylogenetic information into traditional meta-analysis (Lajeunesse, 2009), to address the following questions: Is there any general pattern of biomass allocation of clonal plants (i) between vegetative growth and reproduction, (ii) between aboveground and belowground, (iii) between clonal reproduction and sexual reproduction, in response to change in resources (e.g., light intensity, nutrient level and water availability)? Do they vary among different types of clonal organ? Whether, biomass allocation of clonal plants is genetically determined or responsive to environment?

**Table 1:** **Trait categories**.

**Trait category**	**Trait sub-category**	**Traits**
Vegetative growth (VG)	Aboveground (AG)	Aboveground biomass (or shoot biomass): leaf DW and stem DW
	Belowground (BG)	Belowground biomass (or root biomass): root DW
Reproduction (RE)	Clonal reproduction (CR)	Clonal reproductive biomass (or spacer biomass): rhizome DW or stolon DW
	Sexual reproduction (SR)	Sexual reproductive biomass: flower DW, fruit DW, and seed DW
Vegetative proportion (VGP)	Aboveground proportion (AGP)	Aboveground biomass proportion (or shoot biomass proportion): leaf DWP and stem DWP
	Belowground proportion (BGP)	Belowground biomass proportion (or root biomass proportion): root DWP
Reproductive proportion (REP)	Clonal reproductive proportion (CRP)	Clonal reproductive biomass proportion (or spacer biomass proportion): rhizome DWP or stolon DWP
	Sexual reproductive proportion (SRP)	Sexual reproductive biomass proportion: flowers DWP, fruits DWP, and seeds DWP

*DW, dry weight; DWP, dry weight proportion*.

## Materials and methods

### Literature survey and data selection criteria

To perform a comprehensive literature survey, we conducted an exhaustive search primarily relying on the internet search engine, Google Scholar (Beckmann and von Wehrden, 2012), and supplemented by additional searches based on main databases (i.e., ISI Web of Knowledge, Science Direct, Wiley-Blackwell, Springer Link, CNKI [China National Knowledge Infrastructure], etc.) with keywords of “trade-off,” “biomass allocat^*^” in combination with “clonal plant^*^,” obtained 477 literatures. To identify studies specific to our questions, we did a separate search on papers which referred to “clonal plant^*^,” finally 449 literatures fit the topic of our meta-analysis.

For each literature, we recorded the title, author(s), year, location, and some other information (see the Supplementary Table S1) and examined their potential for meeting the selection criteria for inclusion in review. Foremost, only experimental studies in greenhouse, common garden or field were taken into account, while reviews, models and other studies were excluded. Secondly, we only included studies that reported traits (Table 1) related to biomass allocation strategy in response to resource availability (i.e., light intensity, nutrient level and water availability). Furthermore, we excluded the studies in which the means, standard deviations and sample sizes for the treatment and control group were neither reported nor able to be inferred (or calculated) from other information (Gurevitch et al., 1992). The final data set contained 139 literatures published in 50 journals between 1973 and 2013, from which we extracted data for the meta-analyses (Supplementary S2).

### Data assembly

For each study, we extracted the means, the statistical variation (usually SE or SD) and the sample size values for treatment and control groups for each responsive variable (trait). We regarded multiple results within a single paper as different results from independent studies when they contained different species and/or treatments (Wolf, 1986; Gurevitch et al., 1992; Bolnick and Preisser, 2005; Marczak et al., 2007), while only extracted data once from the same experimental results in different papers (Gurevitch et al., 2001). When the study set up experiments on several treatment levels, each “treatment level” was paired with “control” to calculated effect size firstly and would be pooled later. Resource treatments (light intensity, nutrient level and water availability) used in the studies followed the explanation in Xie et al. (2014). All data were extracted from tables or digitized from graphs with the software GetData v2.22 (http://www.getdata-graph-digitizer.com). A total of 2308 comparisons containing 115 clonal plant species from 87 genera in 33 families were involved in analysis at last. For each comparison, we calculated Hedges' *d* as effect size of experimental treatment (Lajeunesse and Forbes, 2003; van Kleunen et al., 2010). The absolute value of Hedges' *d* showed the magnitude of the treatment impact. Positive or negative *d*-values signified an increase or decrease effect of the treatment, respectively. Zero meant no difference between treatment and control group (Rosenberg et al., 2000).

With regard to the comparisons from experiments on multiple treatment levels, we pooled effect sizes and variances of each trait per species and study by doing a separate meta-analysis to avoid pseudo-replication (see also Leimu et al., 2006; van Kleunen et al., 2010; Song et al., 2013). The pooled mean effect sizes and mean variances were used in the final datasets containing 229 cases in light treatment, 380 cases in nutrient treatment and 93 cases in water treatment. For all analyses, we chosen the random-model setting, as we assumed that differences among comparisons and among studies are not only due to sampling errors but also due to true random variations, as is the rule for ecological data (Gurevitch and Hedges, 2001). All effect size calculations were carried out with the software MetaWin, version 2.1 (Rosenberg et al., 2000).

In order to apply PMA, we created phylogenetic trees (Supplementary Figure S3) with branch lengths through the Phylomatic (http://phylodiversity.net/phylomatic/), with option (Phylomatic tree R20120829 for plants) and Phylocom software (Webb and Donoghue, 2005; Webb et al., 2008), and generated a subset tree for each trait category per species. As the restriction of input files executed on phyloMeta v1.3 software (Lajeunesse, 2011), we pooled again those multiple effect sizes on the same species from different studies. This resulted in only one accumulated weighted effect size and variance for each species within a given trait category on one hand, and inevitably resulted in smaller sample sizes for each trait category on the other hand (N_effect−size_ = N_species_) (Carmona et al., 2011; Nakagawa and Santos, 2012). The pooling was also carried out with a random-effect model on MetaWin, version 2.1 (Rosenberg et al., 2000).

### Data analysis

Before all planned analysis, we explored the possibility of publication bias graphically (funnel plot and normal quantile plot; Wang and Bushman, 1998; Gates, 2002), statistically (Spearman rank correlation test; Begg, 1994), and also by calculating a fail-safe number (Rosenthal, 1991; Rosenberg, 2005). As a result, the funnel plot of effect size vs. sample size showed no skewness; the plot of standardized effect sizes against normal quantiles revealed a straight line; the result of Spearman rank-order correlation test on effect size vs. sample size was not significant (R = 0.050, *P* = 0.187); the fail-safe number 100,896 was much greater than 3520 (i.e., 5n + 10; n was the number of cases in our dataset, Supplementary Table S1). All results of those tests indicated that there was no evidence of publication bias (Supplementary Figures S4, S5).

We categorized each trait into trait category and trait sub-category (Table 1), for example, sexual reproductive biomass, if there was a species with multiple traits, e.g., flower biomass, fruit biomass, we calculated the sum of them. For each trait category, we calculated the overall effect sizes (*d*++) of light, nutrient and water separately on the relevant responsive variables. The overall effect sizes were cumulative effect sizes per species (Hedges and Olkin, 1985; Lajeunesse, 2009). For each part of biomass allocation, we analyzed the response of clonal plants in two aspects: absolute biomass value and its proportion to total value (Table 1). The data might be not independent because the absolute value and relative value could share some data. To detect the differences between stoloniferous plants and rhizomatous plants, we considered the type of clonal organs as moderator variable. In this paper we just concerned the two types of clonal plants. The analyses were performed on the software phyloMeta v1.3 (Lajeunesse, 2011).

## Results

### Biomass allocation between vegetative growth and reproduction of clonal plants

**Figure 1:**
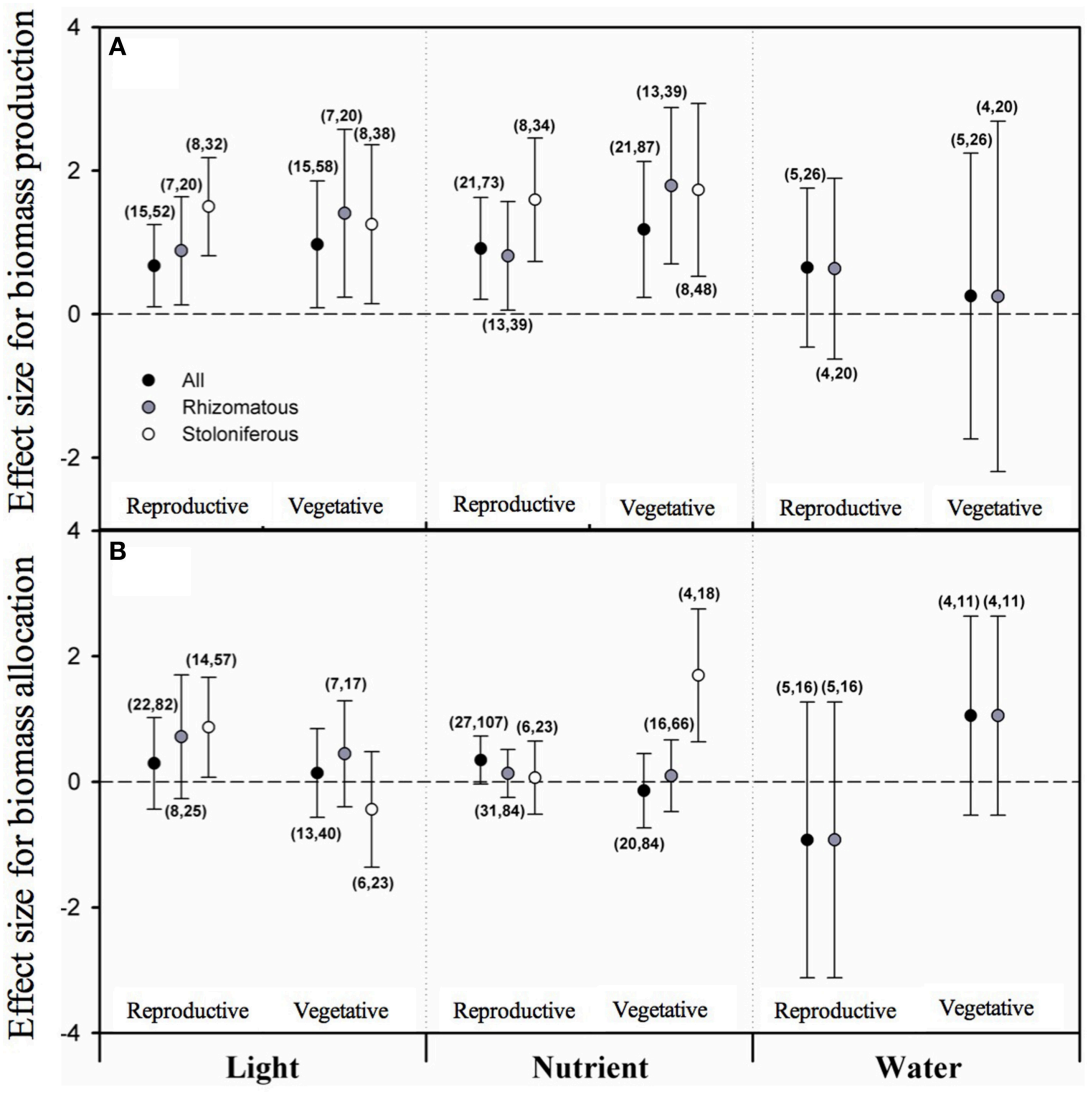
**The mean effect sizes of three types of resources (light, nutrient and water) on biomass production (A) and biomass allocation (B) between vegetative growth and reproduction for all plants (black circles), rhizomatous plants (gray circles), and stoloniferous plants (white circles) from random-model in PMA by software phyloMeta v1.3**. The bars around the means denote bias-corrected 95% bootstrap confidence intervals, and a mean effect size is significantly different from zero when its 95% confidence interval does not include zero. The first and second numbers in brackets are number of species and number of studies, respectively.

According to the PMA results, the overall effect sizes of light intensity on biomass allocated to both vegetative growth (VG) and reproduction (RE) were positive and significantly different from zero, and so did those of nutrient level on VG and RE (Figure 1A). But the grand mean effect size of water availability neither on VG nor on RE was different from zero. Considering clonal organ types, the mean effect sizes of light intensity and nutrient level on VG and RE were still positive and significantly different from zero for both rhizomatous and stoloniferous plants (Figure 1A). The mean effect sizes of water availability on VG and RE of rhizomatous plants were not different from zero. The data of stoloniferous plants were too few to analyze.

From the perspective of biomass proportion, none of the overall effect sizes of light intensity, nutrient level and water availability on biomass proportion allocated to vegetative growth (VGP) or reproduction (REP) were significant (Figure 1B). None of mean effect sizes of rhizomatous plants in response to light intensity, nutrient level and water availability on VGP or REP were significant either. However, the mean effect size of stoloniferous plants in response to light intensity on REP and that of nutrient level on VGP were positive (Figure 1B).

### Biomass allocation between aboveground and belowground part of clonal plants

**Figure 2:**
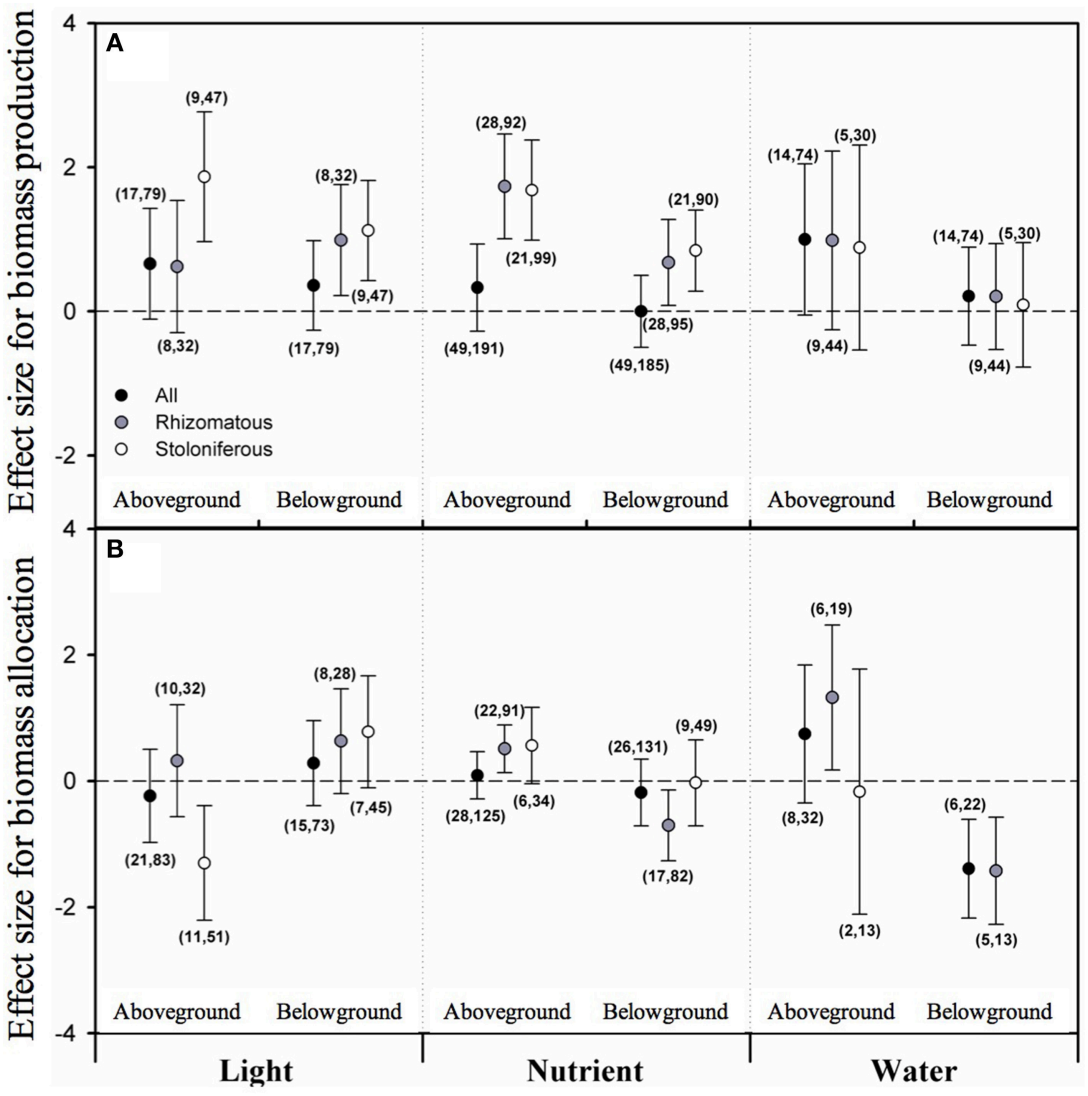
**The the mean effect sizes of three types of resources (light, nutrient, and water) on biomass production (A) and biomass allocation (B) between aboveground and belowground for all plants (black circles), rhizomatous plants (gray circles), and stoloniferous plants (white circles) from random-model in PMA by software phyloMeta v1.3**. The bars around the means denote bias-corrected 95% bootstrap confidence intervals, and a mean effect size is significantly different from zero when its 95% confidence interval does not include zero. The first and second numbers in brackets are number of species and number of studies, respectively.

None of the overall effect sizes of light intensity, nutrient level and water availability on biomass allocated to aboveground (AG) or belowground (BG) was significant irrespective of clonal organ types. Taking clonal organ type into account, however, the mean effect sizes of light intensity and nutrient level on both AG and BG of stoloniferous plants were positive and significant; also positive and significant were the mean effect sizes of light intensity on BG and of nutrient level on AG and BG in rhizomatous plants (Figure 2A). However, the mean effect sizes of water availability on AG and BG in either rhizomatous or stoloniferous plant were not statistically significant (Figure 2A).

In biomass proportion perspective, only the overall effect size of water availability on biomass proportion allocated to belowground (BGP) was significantly negative irrespective of clonal organ types (Figure 2B). For rhizomatous plants, the mean effect sizes of nutrient level and water availability on biomass proportion allocated to aboveground (AGP) were significant and positive and those on BGP were significant and negative, but the effect size of light intensity neither on AGP nor on BGP was significant (Figure 2B). Whereas, only the mean effect size of light intensity on AGP was significant and negative for stoloniferous plants, the mean effect sizes of nutrient level and water availability on AGP or BGP were not significant, and the data were not sufficient to analyze effect of water availability on BGP (Figure 2B).

In addition, we conducted supplementary analyses for the effects of light intensity, nutrient level and water availability on the ratio of root to shoot (R/S), and the results indicated that the effect sizes of light intensity on R/S were significant and positive, for rhizomatous plants: *d*+ = 0.939, *N* = 12, 95% *CI* = [0.257, 1.622] (N: number of species; CI: confidence interval), and for stoloniferous plants: *d*+ = 1.379, *N* = 12, 95% *CI* = [0.615, 2.143]. And the effect sizes of nutrient level on R/S were significant and negative, for rhizomatous plants: *d*+ = −0.891, *N* = 18, 95% *CI* = [−1.608, −0.174], and for stoloniferous plants: *d*+ = −1.272, *N* = 14, 95% *CI* = [−1.885, −0.659]. The effect sizes of water availability on R/S were only significant and negative for stoloniferous plants (*d*+ = −1.082, *N* = 5, 95% *CI* = [−2.057, −0.106]), but not significant for rhizomatous plants.

### Biomass allocation between clonal and sexual reproduction of clonal plants

**Figure 3:**
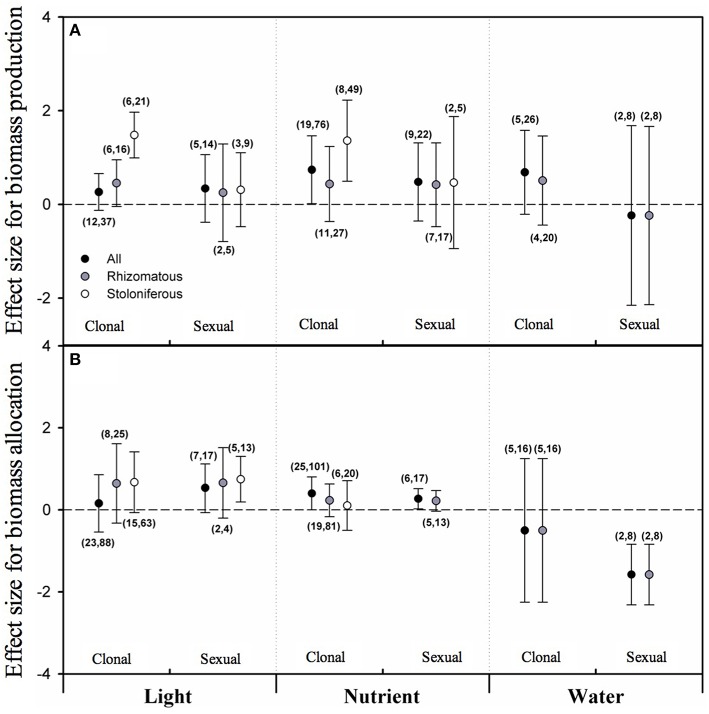
**The mean effect sizes of three types of resources (light, nutrient and water) on biomass production (A) and biomass allocation (B) between clonal reproduction and sexual reproduction for all plants (black circles), rhizomatous plants (gray circles), and stoloniferous plants (white circles) from random-model in PMA by software phyloMeta v1.3**. The bars around the means denote bias-corrected 95% bootstrap confidence intervals, and a mean effect size is significantly different from zero when its 95% confidence interval does not include zero. The first and second numbers in brackets are number of species and number of studies, respectively.

Based on the results of PMA, none of the overall effect sizes of light intensity, nutrient level and water availability on biomass allocated to clonal reproduction (CR) or sexual reproduction (SR) were significant irrespective of clonal organ type (Figure 3A). As taking clonal organ type into account, although the mean effect sizes for rhizomatous plants were not significant yet, the effect sizes of light intensity and nutrient level on CR for stoloniferous plants were significant and positive (Figure 3A). There were not sufficient data for analyze the effect of water availability on CR or SR of stoloniferous plants.

For biomass proportion, the overall effect sizes of nutrient level on biomass proportion allocated to sexual reproduction (SRP) was significant and positive (Figure 3B). Only the mean effect size of light intensity on SRP of stoloniferous plants was significant and positive, and that of water availability on biomass proportion allocated to sexual reproduction (SRP) of rhizomatous plants was significant and negative (Figure 3B). The data were not sufficient to analyze the effects of nutrient level and water availability on SRP of stoloniferous plants and that of water availability on CRP of stoloniferous plants.

## Discussion

Conclusions from individual species or single experiment were highly diverse, depending on species and environmental factors. This paper reported the overall results of PMA to draw the general pattern of biomass allocation in clonal plants in response to resource availability. A coherent picture of some aspects (i.e., growth vs. reproduction, aboveground vs. belowground part, and clonal vs. sexual reproduction) of biomass allocation strategy emerges.

### Biomass allocation to growth vs. reproduction in clonal plants

Growth and reproduction are among the most fundamental activities in plants. Once plant initiates reproductive machinery via growth, its biomass allocation requires investment trade-offs, as resources allocated to one function or organ are unavailable for other functions or organs (Weiner, 2004; Weiner et al., 2009). But the expectation that trade-offs would occur between the competing functions of vegetative growth and reproduction is not upheld in this study. Our results show that clonal plants simultaneously increase biomass allocated to vegetative growth and reproduction with light intensity and nutrient level increased, no matter of the clonal organ types. This is unsurprising and complies with common sense, and also can be regarded as a result of individual growth (Coleman et al., 1994). The results further shown that the biomass proportions allocated to vegetative growth and reproduction of rhizomatous plants neither increased with light intensity, nutrient level or water availability increased, which suggests that changes of resource level had not impact on pattern of biomass allocation between growth and reproduction in rhizomatous plants (Bazzaz et al., 2000). While for stoloniferous plants, the biomass proportion allocated to reproduction increased with the increasing light intensity, and the biomass proportion allocated to vegetative growth increased with the increasing nutrient level. These results imply that stoloniferous plants would decrease biomass proportion allocated to reproduction in shaded environment and decrease biomass proportion allocated to vegetative growth under nutrient-poor conditions. This might be explained as that stoloniferous plants would rather sacrifice reproduction to maintain the vegetative growth to seek light resource, and rather ensure reproduction at the cost of vegetative growth to get away from the infertile habitat. So by comparison, the biomass allocation in rhizomatous plants is much more determined by ontogeny while that of stoloniferous plants is more susceptible to environmental changes.

Regarding to effect of water availability on biomass allocation, our present data on stoloniferous plants are not sufficient to be analyzed. The results of rhizomatous plants indicate that water availability has no significant effects on the biomass allocation. Although, this may be due to the limiting data, similar results have been reported in previous studies (McConnaughay and Coleman, 1999). Explicit and credible conclusions need more experimental studies to test the effect of water availability on biomass allocation of stoloniferous plants.

### Biomass allocation to aboveground vs. belowground in clonal plants

Numerous studies on biomass allocation of plants have focused on aspects of between above- and below-ground biomass (i.e., root vs. shoot). And the mechanisms underlying the observed partitioning responses of plants have always been the debate center (Müller et al., 2000; Poorter and Nagel, 2000; Shipley and Meziane, 2002). Given the ontogenetic drift and optimal allocation theories, biomass allocation was analyzed and interpreted in terms of size (e.g., aboveground biomass) and proportion (e.g., aboveground or belowground biomass to total biomass) in this study. The ontogenetic drift theory stressed preferential allocation to shoot over root as plant grew larger regardless of environmental conditions (Coleman et al., 1994; Müller et al., 2000), which contradicted our results. Our results suggest that with light intensity and nutrient level increased, stoloniferous plants increased aboveground and belowground biomass almost simultaneously, but decreased the biomass allocated to aboveground with light intensity increased, not belowground (McConnaughay and Coleman, 1999). In comparison with stoloniferous plants, the rhizomatous was not sensitive to light intensity and only increased belowground biomass with light intensity increased, but more susceptible to nutrient availability. With nutrient level increased, rhizomatous plants increased aboveground and belowground biomass simultaneously, but increased aboveground biomass proportion and decreased belowground biomass proportion. So we can infer that the opposite is true: with nutrient level decreased, rhizomatous plants would reduce biomass proportion allocated to aboveground and increase biomass proportion allocated to belowground to search the limiting nutrients, which profoundly supports the optimal allocation theory (Poorter and Nagel, 2000; Shipley and Meziane, 2002). With water availability increased, both rhizomatous and stoloniferous plants had no significant changes in terms of size, but the rhizomatous responded in the same way as they did with nutrient level increased (Gonzáles et al., 2008; Huang et al., 2013).

The results of R/S ratio are also compliance with the optimal allocation theory: R/S in both rhizomatous and stoloniferous plant were increased with light intensity, which may be confounded by ontogenetic drift; and R/S were decreased with nutrient level, which is obviously inconsistent with ontogenetic drift in this point, as according to ontogenetic drift theory, the ratios should be increased with increasing individual size irrespective of the nutrient level (Müller et al., 2000). In addition, it should not be ignored that our results from PMA have excluded the confounding effects of phylogeny.

In general, the optimal allocation theory found in many ecological models involving plant biomass allocation, in which biomass is preferentially allocated to the plant part obtaining the resource that is essential but limiting for growth, appears to be a reasonable explanation of the biomass allocation strategies of clonal plants suggested by our results (Poorter and Nagel, 2000; Shipley and Meziane, 2002). In addition, the results of this study also prove that in terms of biomass allocation, stoloniferous plants are more sensitive to light condition, while rhizomatous plants are more sensitive to nutrient condition.

### Biomass allocation to clonal vs. sexual reproduction in clonal plants

Clonal plants possess two modes of reproduction (clonal and sexual), and each mode has its own pros and cons (Jackson et al., 1985; Wu et al., 2010; Barrett, 2015). How resources are divided between two modes of reproduction has been considerable interest to researchers (Willson, 1983; Bazzaz et al., 1987; Lovett Doust, 1989; Reekie, 1991). A trade-off between clonal and sexual reproduction has been commonplace in clonal plants (Watson, 1984; Silvertown et al., 1993; Svenning, 2000; Wu et al., 2010). This prediction derives from assumption that allocation among competing functions is mutually exclusive, as a plant has a given amount of resources at any point in time, so different allocation patterns reflect different adaptive strategies of clonal plant in response to variable environment (Sutherland and Vickery, 1988; Weiner, 2004; Liu et al., 2009). Some previous studies confounded clonal reproduction and vegetative growth, which might confuse the real trade-off between clonal and sexual reproduction. However, when separated clonal reproduction from vegetative growth, the trade-off patterns of biomass allocation between clonal and sexual reproduction were not detected in this study (Reekie, 1991). Our results exhibit that increasing light intensity, nutrient level and water availability had no significant effect on biomass allocated to clonal and sexual reproduction of rhizomatous plants, as well as biomass proportion. Only one exception to these trends was that biomass proportion allocated to sexual reproduction of the rhizomatous decreased with water availability increased, which means that the rhizomatous preferentially allocate biomass to the sexual reproduction under low-water condition (Li et al., 2001b). This is in line with the notion that sexual reproduction may allow escape from poor conditions and produce genetically diverse offspring that may be better able to cope with harsh conditions (Eriksson, 1997; Gardner and Mangel, 1999; van Kleunen et al., 2002). As to stoloniferous plants, high light intensity and nutrient level resulted in increased biomass of clonal reproduction without concomitant decreases of sexual reproduction, and inversely high light intensity resulted in increased biomass proportion of sexual reproduction without concomitant decreases of clonal reproduction. The former can be explained as that clonal reproduction may be beneficial as a means to remain in benign environments (Abrahamson, 1975; van Kleunen et al., 2002), and the later one is consistent with the earlier result of this study that stoloniferous plants would rather sacrifice reproduction to maintain the vegetative growth to capture light resource in shaded environment (Svenning, 2000). Therefore, these results reveal that biomass allocation of clonal plants to reproduction is much more constrained by ontogeny or heredity than by environments, and that stoloniferous plants are relatively more susceptible to environments than rhizomatous plants in biomass allocation between clonal and sexual reproduction.

According to current results, there might be no trade-offs between vegetative growth and reproduction, clonal and sexual reproduction in biomass allocation of clonal plants (Pitelka et al., 1985; Reekie, 1991; Mendoza and Franco, 1998; Svenning, 2000). The two critical preconditions of trade-off are that the resource is in fixed supply and that allocation among competing functions is mutually exclusive (Watson, 1984; Bazzaz et al., 2000). But in its application to the study of reproductive strategies, these two preconditions may be not always true for two principal reasons. Firstly, some processes, such as the consecutive photosynthesis of plants, can lead to an increase in total resource supply associated with reproduction, because light, nutrients and other resources are supplied continuously. Secondly, plant structures can contribute to more than one function and may well not be mutually exclusive (Bazzaz et al., 2000). As a result, measures of allocation to different structures or functions do not always exhibit the trade-off patterns.

In conclusion, our study used PMA to analyze the response of functional traits related to biomass allocation of clonal plant to changing environments. Here, we summarize several general patterns based on the PMA results: (i) clonal plants exhibit higher plasticity of vegetative growth traits than reproduction traits in response to resource levels; (ii) in response to resources constrains biomass allocation patterns between belowground and aboveground parts of clonal plants conform to optimal allocation theory; (iii) no evidence was found of trade-off patterns between clonal and sexual reproduction. All biomass allocation strategies of clonal plants obey the tenet that tends to maximize genet fitness, whether conforming to optimal allocation theory or constrained by ontogeny. The optimal allocation theory explained the “true plasticity” of clonal plants to cope with changing environments, while the ontogeny drift theory emphasized the genetic influence on plant growth. In this paper, we just tried to clarify the trade-off strategies of clonal plants in changing environments using PMA method with which phylogenetic effect was avoided. Our results profoundly support the optimal allocation theory rather than disprove the ontogeny drift theory. Besides, this study analyzed trade-off strategies in terms of biomass allocation in clonal plants, but allocations of some other resources, such as meristems (Bazzaz et al., 2000) were not considered due to data limitation. Meanwhile, the results related to water availability must be interpreted with caution because the outcomes are perhaps caused by data shortage. So we highlight that experimental studies are essential and indispensable whatsoever in the past, present or future.

## Author contributions

Conceived and designed the experiments: MD, YS. Performed the experiments: XX, YH. Contributed to the writing of the manuscript: XX, YH, XP, FL, YS, and MD.

## Funding

The funder had no role in study design, data collection and analysis, decision to publish, or preparation the manuscript.

### Conflict of interest statement

The authors declare that the research was conducted in the absence of any commercial or financial relationships that could be construed as a potential conflict of interest.
