# Supplementary material for: Biomass Allocation of Stoloniferous and Rhizomatous Plant in Response to Resource Availability: A Phylogenetic Meta-Analysis
Source: Front Plant Sci. 2016 May 4;7:603. doi: 10.3389/fpls.2016.00603 (PMC4854891; doi:10.3389/fpls.2016.00603)
Supplement: Supplementary file 2 [file Table2.DOC]

**Supplementary S2 Literature list used in meta-analysis**

Abrahamson W.G. & McCrea K.D. (1986). Nutrient and biomass allocation in *Solidago altissima*: Effects of two stem gallmakers, fertilization, and ramet isolation. *Oecologia*, 68, 174-180.

Alpert P. (1991). Nitrogen sharing among ramets increases clonal growth in *Fragaria chiloensis*. *Ecology*, 72, 69-80.

Alpert P. (1996). Nutrient sharing in natural clonal fragments of *Fragaria chiloensis*. *J Ecol*, 84, 395-406.

Alpert P. (1999). Clonal integration in *Fragaria chiloensis* differs between populations: Ramets from grassland are selfish. *Oecologia*, 120, 69-76.

Alpert P. & Mooney H.A. (1986). Resource sharing among ramets in the clonal herb, *Fragaria chiloensis*. *Oecologia*, 70, 227-233.

Ba L., Wang D.L., Hodgkinson K.C. & Xiao N.Z. (2006). Competitive relationships between two contrasting but coexisting grasses. *Plant Ecol*, 183, 19-26.

Bai W.M., Sun X.Q., Wang Z.W. & Li L.H. (2009). Nitrogen addition and rhizome severing modify clonal growth and reproductive modes of *Leymus chinensis* population. *Plant Ecol*, 205, 13-21.

Barko J.W., Hardin D.G. & Matthews M.S. (1982). Growth and morphology of submersed freshwater macrophytes in relation to light and temperature. *Can J Bot*, 60, 877-887.

Bartoš M., Janeček Š. & Klimešová J. (2011). Effect of mowing and fertilization on biomass and carbohydrate reserves of *Molinia caerulea* at two organizational levels. *Acta Oecol*, 37, 299-306.

Caradus J.R. (1992). Heritability of, and relationships between phosphorus and nitrogen concentration in shoot, stolon and root of white clover (*Trifolium repens* L.). *Plant Soil*, 146, 209-217.

Carter E.B., Theodorou M.K. & Morris P. (1997). Responses of Lotus corniculatus to environmental change .1. Effects of elevated CO_2_, temperature and drought on growth and plant development. *New Phytol*, 136, 245-253.

Chapman D., Robson M. & Snaydon R. (1992). Interactions between defoliation and the nitrogen nutrition of white clover (*Trifolium repens* L.): Effects on carbon utilisation in clonal plants. *Plant Soil*, 139, 157-165.

Chen J.S. (2004). Study on the adaptive strategies of clonal plants in heterogeneous environments. In. Wuhan University Wuhan, p. 118.

Chen J.S., Lei N.F., Yu D. & Dong M. (2006). Differential effects of clonal integration on performance in the stoloniferous herb *Duchesnea indica*, as growing at two sites with different altitude. *Plant Ecol*, 183, 147-156.

Cheplick G.P. (1995). Life-history trade-offs in *Amphibromus scabrivalvis* (Poaceae): Allocation to clonal growth, storage, and cleistogamous reproduction. *Am J Bot*, 82, 621-629.

Chu Y., Yu F.H. & Dong M. (2006). Clonal plasticity in response to reciprocal patchiness of light and nutrients in the stoloniferous herb *Glechoma longituba* L. *J Integr Plant Biol*, 48, 400-408.

Claridge K. & Franklin S.B. (2002). Compensation and plasticity in an invasive plant species. *Biol Invasions*, 4, 339-347.

Clevering O.A. (1999). The effects of litter on growth and plasticity of *Phragmites australis* clones originating from infertile, fertile or eutrophicated habitats. *Aquat Bot*, 64, 35-50.

Cronin G. & Lodge D.M. (2003). Effects of light and nutrient availability on the growth, allocation, carbon/nitrogen balance, phenolic chemistry, and resistance to herbivory of two freshwater macrophytes. *Oecologia*, 137, 32-41.

D'Hertefeldt T., Falkengren-Grerup U. & Jonsdottir I.S. (2011). Responses to mineral nutrient availability and heterogeneity in physiologically integrated sedges from contrasting habitats. *Plant Biol*, 13, 483-92.

Dong M. & Alaten B. (1999). Clonal plasticity in response to rhizome severing and heterogeneous resource supply in the rhizomatous grass *Psammochloa villosa* in an Inner Mongolian dune, China. *Plant Ecol*, 141, 53-58.

Dong M., During H.J. & Werger M.J.A. (1997). Clonal plasticity in response to nutrient availability in the pseudoannual herb, *Trientalis europaea* L. *Plant Ecol*, 131, 233-239.

Dong M., During H.J. & Werger M.J.A. (2002). Root and shoot plasticity of the stoloniferous herb *Ajuga reptans* L. planted in a heterogeneous environment. *Flora*, 197, 37-46.

Dong M., Zhang S.M. & Chen Y.F. (2000). Clonal plasticity in response to nutrient availability in the stoloniferous herb, *Duchesnea indica*. *Acta Bot Sin*, 42, 518-522.

Fransen B., de Kroon H. & Berendse F. (1998). Root morphological plasticity and nutrient acquisition of perennial grass species from habitats of different nutrient availability. *Oecologia*, 115, 351-358.

Friedman D. & Alpert P. (1991). Reciprocal transport between ramets increases growth of *Fragaria chiloensis* when light and nitrogen occur in separate patches but only if patches are rich. *Oecologia*, 86, 76-80.

Gebauer R.L.E., Reynolds J.F. & Tenhunen J.D. (1995). Growth and allocation of the arctic sedges *Eriohorum angustifolium* and *E. vaginatum*: Effects of variable soil oxygen and nutrient availability. *Oecologia*, 104, 330-339.

Gianoli E. & González-Teuber M. (2005). Environmental heterogeneity and population differentiation in plasticity to drought in *Convolvulus Chilensis* (Convolvulaceae). *Evol Ecol*, 19, 603-613.

Ginzo H.D. & Lovell P.H. (1973). Aspects of the comparative physiology of *Ranunculus bulbosus* L. and *Ranunculus repens* L. I. Response to nitrogen. *Ann Bot*, 37, 753-764.

Guglielmini A.C. & Satorre E.H. (2002). Shading effects on spatial growth and biomass partitioning of *Cynodon dactylon*. *Weed Res*, 42, 123-134.

Guo W., Song Y.B. & Yu F.H. (2011). Heterogeneous light supply affects growth and biomass allocation of the understory fern *Diplopterygium glaucum* at high patch contrast. *PLoS One*, 6, e27998.

Hartnett D.C. (1993). Regulation of clonal growth and dynamics of *Panicum virgatum* (Poaceae) in tallgrass prairie: Effects of neighbor removal and nutrient addition. *Am J Bot*, 80, 1114-1120.

He W.M., Alpert P., Yu F.H., Zhang L.L. & Dong M. (2011). Reciprocal and coincident patchiness of multiple resources differentially affect benefits of clonal integration in two perennial plants. *J Ecol*, 99, 1202-1210.

He Y.L., Wang M.T., Wen S.J., Zhang Y.H., Ma T. & Du G.Z. (2007a). Seed size effect on seedling growth under different light conditions in the clonal herb *Ligularia virgaurea* in Qinghai-Tibet Plateau. *Acta Ecol Sin*, 27, 3091-3108.

He Z.S., He W.M., Yu F.H., Shi P.L., Zhang X.Z., He Y.T., Zhong Z.M. & Dong M. (2007b). Do clonal growth form and habitat origin affect resource-induced plasticity in Tibetan alpine herbs? *Flora*, 202, 408-416.

Holdredge C., Bertness M.D., von Wettberg E. & Silliman B.R. (2010). Nutrient enrichment enhances hidden differences in phenotype to drive a cryptic plant invasion. *Oikos*, 119, 1776-1784.

Huber H. (1996). Plasticity of internodes and petioles in postrate and erect *Potentilla* species. *Funct Ecol*, 10, 401-409.

Huber H., Fijan A. & During H.J. (1998). A comparative study of spacer plasticity in erect and stoloniferous herbs. *Oikos*, 81, 576-586.

Huber H. & Hutchings M.J. (1997). Differential response to shading in orthotropic and plagiotropic shoots of the clonal herb *Glechoma hirsuta*. *Oecologia*, 112, 485-491.

Huber H., Whigham D.F. & O’neill J. (2005). Timing of disturbance changes the balance between growth and survival of parent and offspring ramets in the clonal forest understory herb *Uvularia perfoliata*. *Evol Ecol*, 18, 521-539.

Huber H. & Wiggerman L. (1997). Shade avoidance in the clonal herb Trifolium fragiferum/: a field study with experimentally manipulated vegetation height. *Plant Ecol*, 130, 53-62.

Huffman D.W., Zasada J.C. & Tappeiner Ii J.C. (1994). Growth and morphology of rhizome cuttings and seedlings of salal (*Gaultheria shallon*): Effects of four light intensities. *Can J Bot*, 72, 1702-1708.

Humphrey L.D. & Pyke D.A. (1997). Clonal foraging in perennial wheatgrasses: A strategy for exploiting patchy soil nutrients. *J Ecol*, 85, 601-610.

Ikegami M., Whigham D. & Werger M. (2007). Responses of rhizome length and ramet production to resource availability in the clonal sedge *Scirpus olneyi* A. Gray. *Plant Ecol*, 189, 247-259.

Ikegami M., Whigham D. & Werger M. (2009). Ramet phenology and clonal architectures of the clonal sedge *Schoenoplectus americanus* (Pers.) Volk. ex Schinz & R. Keller. *Plant Ecol*, 200, 287-301.

James J. (2008). Effect of soil nitrogen stress on the relative growth rate of annual and perennial grasses in the Intermountain West. *Plant Soil*, 310, 201-210.

Jia X., Yang X.Z., Pan X.Y., Li B. & Chen J.K. (2008). Vegetative propagation characteristics of *Alternanthera philoxeroides* in response to disturbances. *Biodiversity Science*, 16, 229-235.

Jongejans E., de Kroon H. & Berendse F. (2006). The interplay between shifts in biomass allocation and costs of reproduction in four grassland perennials under simulated successional change. *Oecologia*, 147, 369-78.

Kang X.Y. (2007). Study on the growth character in the clonal plant: *Potentilla anserine* L. In. Qinghai University Xining, p. 49.

Kemball W.D., Palmer M.J. & Marshall C. (1992). The effect of local shading and darkening on branch growth, development and survival in *Trifolium repens* and *Galium aparine*. *Oikos*, 63, 366-375.

Klimeš L. & Klimešová J. (2001). The effects of mowing and fertilization on carbohydrate reserves and regrowth of grasses: Do they promote plant coexistence in species-rich meadows? *Evol Ecol*, 15, 363-382.

Li B., Shibuya T., Yogo Y. & Hara T. (2004). Effects of ramet clipping and nutrient availability on growth and biomass allocation of yellow nutsedge. *Ecol Res*, 19, 603-612.

Li B., Shibuya T., Yogo Y., Hara T. & Matsuo K. (2001a). Effects of light quantity and quality on growth and reproduction of a clonal sedge, *Cyperus esculentus*. *Plant Species Biol*, 16, 69-81.

Li B., Shibuya T., Yogo Y., Hara T. & Yokozawa M. (2001b). Interclonal differences, plasticity and trade-offs of life history traits of *Cyperus esculentus* in relation to water availability. *Plant Species Biol*, 16, 193-207.

Li D.Z., Shigeo T. & Zhu T.C. (2006a). Effects of clonal connection and segmentation on the primary stolons of *Zoysia japonica* in environments with heterogeneous soil nitrogen resources. *Acta Pratacult Sin*, 15, 115-123.

Li J., Ma X.F., Guo P. & Bao G.Z. (2006b). Effects of fertilizer and auxin supply on clonal architectures of *Leymus chinensis*. *Pratacult Sci*, 23, 18-21.

Li Q.Y. (2006). Effects of different soil nutrients on clonal growth and sexual reproduction in *Iris Japonica* Thunb. In. Southwest University Chongqing, p. 47.

Li W.G. & Wang J.B. (2011). Influence of light and nitrate assimilation on the growth strategy in clonal weed *Eichhornia crassipes*. *Aquat Ecol*, 45, 1-9.

Li W.G., Wang J.L., Shen J.J. & Wang J.B. (2009). Nitrate reduction in ramets of a clonal plant *Eichhornia crassipes* responding to nitrate availability during clonal growth stage. *Biol Plant*, 53, 171-174.

Liu C.H. (2006). Study on the invasion ecology of exotic species *Alternanthera philoxeroides* (Mart.) Griseb. In. Wuhan University Wuhan, p. 119.

Liu F., Wu W.Y., Wan T., Wang Q.F., Cheng Y. & Li W. (2013). Temporal variation of resource allocation between sexual and asexual structures in response to nutrient and water stress in a floating-leaved plant. *J Plant Ecol,* 6, 499-505.

Liu Q., Li Y.X. & Zhong Z.C. (2004). Effects of moisture availability on clonal growth in bamboo *Pleioblastus maculata*. *Plant Ecol*, 173, 107-113.

Liu Y., Schieving F., Stuefer J.F. & Anten N.P. (2007). The effects of mechanical stress and spectral shading on the growth and allocation of ten genotypes of a stoloniferous plant. *Ann Bot*, 99, 121-30.

Luo W.B. & Xie Y.H. (2009). Growth and morphological responses to water level and nutrient supply in three emergent macrophyte species. *Hydrobiologia*, 624, 151-160.

Müller I., Schmid B. & Weiner J. (2000). The effect of nutrient availability on biomass allocation patterns in 27 species of herbaceous plants. *Persp Plant Ecol Evol Syst*, 3, 115-127.

Méthy M., Alpert P. & Roy J. (1990). Effects of light quality and quantity on growth of the clonal plant *Eichhornia crassipes*. *Oecologia*, 84, 265-271.

Mao W., Zhang T.H., Li Y.L., Zhao X.Y. & Huang Y.X. (2012). Allometric response of perennial *Pennisetum centrasiaticum* Tzvel to nutrient and water limitation in the Horqin Sand Land of China. *J Arid Land*, 4, 161-170.

Marshall C. & Anderson-Taylor G. (1992). Mineral nutritional inter-relations amongst stolons and tiller ramets in *Agrostis stolonifera* L. *New Phytol*, 122, 339-347.

Martina J.P. & Ende C.N. (2013). Increased spatial dominance in high nitrogen, saturated soil due to clonal architecture plasticity of the invasive wetland plant, *Phalaris arundinacea*. *Plant Ecol*, 214, 1443-1453.

Martina J.P. & von Ende C.N. (2012). Highly plastic response in morphological and physiological traits to light, soil-N and moisture in the model invasive plant, *Phalaris arundinacea*. *Environ Exp Bot*, 82, 43-53.

Maurer D.A. & Zedler J.B. (2002). Differential invasion of a wetland grass explained by tests of nutrients and light availability on establishment and clonal growth. *Oecologia*, 131, 279-288.

Miao S.L. (2004). Rhizome growth and nutrient resorption: Mechanisms underlying the replacement of two clonal species in Florida Everglades. *Aquat Bot*, 78, 55-66.

Navas M.L. & Garnier E. (2002). Plasticity of whole plant and leaf traits in *Rubia peregrina* in response to light, nutrient and water availability. *Acta Oecol*, 23, 375-383.

Nicholls A.M. (2011). Influences of environmental variability, genetics and plant size on variation in sexual and clonal reproduction and allocation of resources in three wetland plant species. In: *Biological, Geological and Environmental Sciences*. Cleveland State University, p. 143.

Nilsson J. & D'Hertefeldt T. (2008). Origin matters for level of resource sharing in the clonal herb *Aegopodium podagraria*. *Evol Ecol*, 22, 437-448.

Niva M., Svensson B.M. & Karlsson P.S. (2006). Effects of light and water availability on shoot dynamics of the stoloniferous plant *Linnaea borealis*. *Ecoscience*, 13, 318-323.

Olga A C. (1998). An investigation into the effects of nitrogen on growth and morphology of stable and die-back populations of *Phragmites australis*. *Aquat Bot*, 60, 11-25.

Peterson A.G. & Chesson P. (2002). Short-term fitness benefits of physiological integration in the clonal herb *Hydrocotyle peduncularis*. *Austr Ecol*, 27, 647-657.

Powelson R.A. & Lieffers V.J. (1992). Effect of light and nutrients on biomass allocation in *Calamagrostis canadensis*. *Ecography*, 15, 31-36.

Qian Y.Q., Luo D., Gong G., Han L., Ju G.S. & Sun Z.Y. (2014). Effects of spatial scale of soil heterogeneity on the growth of a clonal plant producing both spreading and clumping ramets. *J Plant Growth Reg*, 33, 214-221.

Roiloa S.R. & Hutchings M.J. (2013). The effects of physiological integration on biomass partitioning in plant modules: An experimental study with the stoloniferous herb *Glechoma hederacea*. *Plant Ecol*, 214, 521-530.

Roiloa S.R. & Retuerto R. (2007). Responses of the clonal *Fragaria vesca* to microtopographic heterogeneity under different water and light conditions. *Environ Exp Bot*, 61, 1-9.

Saitoh T., Seiwa K. & Nishiwaki A. (2002). Importance of physiological integration of dwarf bamboo to persistence in forest understorey: A field experiment. *J Ecol*, 90, 78-85.

Saitoh T., Seiwa K. & Nishiwaki A. (2006). Effects of resource heterogeneity on nitrogen translocation within clonal fragments of *Sasa palmata*: An isotopic (^15^N) assessment. *Ann Bot*, 98, 657-663.

Schmid B., Miao S. & Bazzaz F. (1990). Effects of simulated root herbivory and fertilizer application on growth and biomass allocation in the clonal perennial *Solidago canadensis*. *Oecologia*, 84, 9-15.

Shan B.Q., Du G.Z. & Liu Z.H. (2000). Clonal growth of *Ligularia virgaurea*: Morphological response to nutritional variation. *Acta Phytoecol Sin*, 24, 46-51.

Shaver G.R., Iii F.C. & Gartner B.L. (1986). Factors limiting seasonal growth and peak biomass accumulation in *Eriophorum vaginatum* in Alaskan Tussock Tundra. *J Ecol*, 74, 257-278.

Shibaike H., Ishiguri Y. & Kawano S. (1996). Plastic responses to nutrient and light intensity gradients in populations of *Oxalis corniculata* L. (Oxalidaceae). *Plant Species Biol*, 11, 213-223.

Slade A.J. & Hutchings M.J. (1987a). Clonal integration and plasticity in foraging behavior in *Glechoma hederacea*. *J Ecol*, 75, 1023-1036.

Slade A.J. & Hutchings M.J. (1987b). The effects of light intensity on foraging in the clonal herb *Glechoma hederacea*. *J Ecol*, 75, 639-650.

Slade A.J. & Hutchings M.J. (1987c). The effects of nutrient availability on foraging in the clonal herb *Glechoma hederacea*. *J Ecol*, 75, 95-112.

Stuefer J.F., During H.J. & de Kroon H. (1994). High benefits of clonal integration in two stoloniferous species, in response to heterogeneous light environments. *J Ecol*, 82, 511-518.

Sun X.L., Niu J.Z., Xu Y.F. & Zhou H. (2010). Long term water integration in interconnected ramets of stoloniferous grass, buffalograss. *Afr J Biotech*, 9, 5503-5510.

Sun X.L., Niu J.Z. & Zhou H. (2011). Buffalograss decreases ramet propagation in infertile patches to enhance interconnected ramet proliferation in fertile patches. *Flora*, 206, 380-386.

Tao J.P. & Zhong Z.C. (2000). Morphological responses to different nutrient supply in the stoloniferous herb *Glechoma longituba*. *Acta Ecol Sin*, 2, 207-211.

Thomas R. & Hay M. (2008). Adaptive variation in physiological traits underpinning stem elongation responses among nodally-rooting stoloniferous herbs. *Evol Ecol*, 22, 369-381.

Thompson F.L. & Eckert C.G. (2004). Trade-offs between sexual and clonal reproduction in an aquatic plant: Experimental manipulations vs. phenotypic correlations. *J Evol Biol*, 17, 581-592.

van Staalduinen M.A. & Anten N.P. (2005). Differences in the compensatory growth of two co-occurring grass species in relation to water availability. *Oecologia*, 146, 190-9.

Verburg R. & Grava D. (1998). Differences in allocation patterns in clonal and sexual offspring in a woodland pseudo-annual. *Oecologia*, 115, 472-477.

Verburg R.W. & During H.J. (1998). Vegetative propagation and sexual reproduction in the woodland understorey pseudo-annual *Circaea lutetiana* L. *Plant Ecol*, 134, 211-224.

Verburg R.W., Kwant R. & Werger M.J.A. (1996). The effect of plant size on vegetative reproduction in a pseudo-annual. *Vegetatio*, 125, 185-192.

Vojtíšková L., Munzarová E., Votrubová O., Řihová A. & Juřicová B. (2004). Growth and biomass allocation of sweet flag (*Acorus calamus* L.) under different nutrient conditions. *Hydrobiologia*, 518, 9-22.

Wang J.C., Shi X., Yin L.K. & Zhang D.Y. (2011). Role of clonal integration in life strategy of sandy dune plant, *Eremosparton Songoricum* (Litv.) Vass (Fabaceae): Experimental approach. *Polish J Ecol*, 59, 455-461.

Wang J.W. & Yu D. (2007). Influence of sediment fertility on morphological variability of *Vallisneria spiralis* L. *Aquat Bot*, 87, 127-133.

Wang M.T., Zhao Z.G., Du G.Z. & He Y.L. (2008a). Effects of light on the growth and clonal reproduction of *Ligularia virgaurea*. *J Integr Plant Biol*, 50, 1015-23.

Wang R.Z., Chen L., Bai Y.G. & Xiao C.W. (2008b). Seasonal dynamics in resource partitioning to growth and storage in response to drought in a perennial rhizomatous grass, *Leymus chinensis*. *J Plant Growth Reg*, 27, 39-48.

Wang Y.J., Shi X.P. & Zhong Z.C. (2013). The relative importance of sexual reproduction and clonal propagation in rhizomatous herb *Iris japonica* Thunb. from two habitats of Jinyun Mountain, Southwest China. *Russ J Ecol*, 44, 199-206.

Weigelt A., Steinlein T. & Beyschlag W. (2005). Competition among three dune species: The impact of water availability on below–ground processes. *Plant Ecol*, 176, 57-68.

Wesser S.D. (1991). The effects of light and moisture on two species from contiguous communities of south-facing bluffs in Interior Alaska, U.S.A. *Arctic Alpine Res*, 23, 99-103.

Wijesinghe D.K. & Hutchings M.J. (1996). Consequences of patchy distribution of light for the growth of the clonal herb *Glechoma hederacea*. *Oikos*, 77, 137-145.

Wijesinghe D.K. & Hutchings M.J. (1997). The effects of spatial scale of environmental heterogeneity on the growth of a clonal plant: An experimental study with glechoma hederacea. *J Ecol*, 85, 17-28.

Wijesinghe D.K. & Whigham D.F. (2001). Nutrient foraging in woodland herbs: A comparison of three species of *Uvularia* (Liliaceae) with contrasting belowground morphologies. *Am J Bot*, 88, 1071-9.

Wolfer S.R. (2008). Clonal architecture and patch formation of *Potamogeton perfoliatus* L. in response to environmental conditions. In. Wageningen Universiteit, p. 120.

Wolfer S.R. & Straile D. (2004). Spatio-temporal dynamics and plasticity of clonal architecture in *Potamogeton perfoliatus*. *Aquat Bot*, 78, 307-318.

Wolfer S.R. & Straile D. (2012). To share or not to share: Clonal integration in a submerged macrophyte in response to light stress. *Hydrobiologia*, 684, 261-269.

Woo I. & Zedler J. (2002). Can nutrients alone shift a sedge meadow towards dominance by the invasive *Typha × glauca*. *Wetlands*, 22, 509-521.

Xiao K., Yu D. & Wang J. (2006). Habitat selection in spatially heterogeneous environments: A test of foraging behaviour in the clonal submerged macrophyte *Vallisneria spiralis*. *Freshwater Biol*, 51, 1552-1559.

Xiao K.Y., Yu D., Xu X.W. & Xiong W. (2007). Benefits of clonal integration between interconnected ramets of *Vallisneria spiralis* in heterogeneous light environments. *Aquat Bot*, 86, 76-82.

Xie D. & Yu D. (2011). Size-related auto-fragment production and carbohydrate storage in auto-fragment of *Myriophyllum spicatum* L. in response to sediment nutrient and plant density. *Hydrobiologia*, 658, 221-231.

Xie Y.H., Wen M.Z., Yu D. & Li Y.K. (2004). Growth and resource allocation of water hyacinth as affected by gradually increasing nutrient concentrations. *Aquat Bot*, 79, 257-266.

Xing M.J. (2010). The strategy of spacial expansion of clonal plant *Hierochloe glabra* Trin. under homogeneous and heterogeneous habitats. In. Northeast Normal University Jilin, p. 40.

Xu C.Y., Schooler S.S. & Van Klinken R.D. (2012). Differential influence of clonal integration on morphological and growth responses to light in two invasive herbs. *PLoS One*, 7, e35873.

Xu G.F., Shen S.C., Zhang F.D., Li T.L. & Zhang Y.H. (2013). Effect of soil-water conditions on survival rate and morphological plasticity of clonal plant *Mikania micrantha* H.B. Kunth. *Sci Agricult Sin*, 46, 3134-3141.

Xu K.Y., Ye W.H., Li J. & Li G.M. (2005). Phenotypic plasticity in response to soil nutrients in the invasive species *Alternanthera philoxeroides*. *Ecol Environ*, 14, 723-726.

Ye X.H., Yu F.H. & Dong M. (2006). A trade-off between guerrilla and phalanx growth forms in *Leymus secalinus* under different nutrient supplies. *Ann Bot*, 98, 187-191.

You W.H., Yu D., Liu C.H., Xie D. & Xiong W. (2013). Clonal integration facilitates invasiveness of the alien aquatic plant *Myriophyllum aquaticum* L. under heterogeneous water availability. *Hydrobiologia*, 718, 27-39.

Yu F.H. & Dong M. (2003). Effect of light intensity and nutrient availability on clonal growth and clonal morphology of the stoloniferous herb *Halerpestes ruthenica*. *Acta Bot Sin*, 45, 408-416.

Yue C.L., Chang J., Wang K.H. & Zhu Y.M. (2004). Response of clonal growth in *Phyllostachys praecox* f. *prevernalis* to changing light intensity. *Austr J Bot*, 52, 171-174.

Zhang C.Y., Yu F.H., Chen Y.F. & Dong M. (2003). Phenotypic plasticity in response to the heterogeneous water supply in the rhizomatous grass species, *Calamagrostis epigejos* in the Mu Us Sandy Land of China. *Acta Bot Sin*, 45, 1210-1217.

Zhang L.L. & He W.M. (2009). Spatial covariance in resources affects photosynthetic rate and water potential, but not the growth of *Glechoma longituba* fragments. *Flora*, 204, 628-634.

Zhang X.Q., Liu J., Welham C.V.J., Liu C.C., Li D.N., Chen L. & Wang R.Q. (2006). The effects of clonal integration on morphological plasticity and placement of daughter ramets in black locust (*Robinia pseudoacacia*). *Flora*, 201, 547-554.

Zhang Y.C. & Zhang Q.Y. (2013). Clonal integration of *Fragaria orientalis* in reciprocal and coincident patchiness resources: Cost-benefit analysis. *PLoS One*, 8, e80623.

Zhang Y.C., Zhang Q.Y., Yirdaw E., Luo P. & Wu N. (2008). Clonal integration of *Fragaria orientalis* driven by contrasting water availability between adjacent patches. *Bot Stud*, 49, 373-383.

Zhang Z.H., Rengel Z. & Meney K. (2007). Growth and resource allocation of *Canna indica* and *Schoenoplectus validus* as affected by interspecific competition and nutrient availability. *Hydrobiologia*, 589, 235-248.

Zhao C.F., Li H.L. & Luo F.L. (2012). Effects of light heterogeneity on growth of a submerged clonal macrophyte. *Plant Species Biol*, 28, 156-164.

Zhao W., Chen S.P. & Lin G.H. (2008). Compensatory growth responses to clipping defoliation in *Leymus chinensis* (Poaceae) under nutrient addition and water deficiency conditions. *Plant Ecol*, 196, 85-99.

Zhao Y., Qing H., Zhao C., Zhou C., Zhang W., Xiao Y. & An S. (2010). Phenotypic plasticity of *Spartina alterniflora* and *Phragmites australis* in response to nitrogen addition and intraspecific competition. *Hydrobiologia*, 637, 143-155.

Zheng Y.L., Feng Y.L., Liu W.X. & Liao Z.Y. (2009). Growth, biomass allocation, morphology, and photosynthesis of invasive *Eupatorium adenophorum* and its native congeners grown at four irradiances. *Plant Ecol*, 203, 263-271.

Zhu Y.J., Alaten B., Dong M. & Huang Z.Y. (2007). Effects of increasing water or nutrient supplies on reproducuon trade-offs in ttie natural populations of clonal plant, *Hedysarum laeve*. *J Plant Ecol*, 31, 658-664.
