# Supplementary material for: Biomass Allocation of Stoloniferous and Rhizomatous Plant in Response to Resource Availability: A Phylogenetic Meta-Analysis
Source: Front Plant Sci. 2016 May 4;7:603. doi: 10.3389/fpls.2016.00603 (PMC4854891; doi:10.3389/fpls.2016.00603)
Supplement: Supplementary file 3 [file Image1.PDF]

Supplementary Figure S3 Phylogenetic trees and Newick file of the species

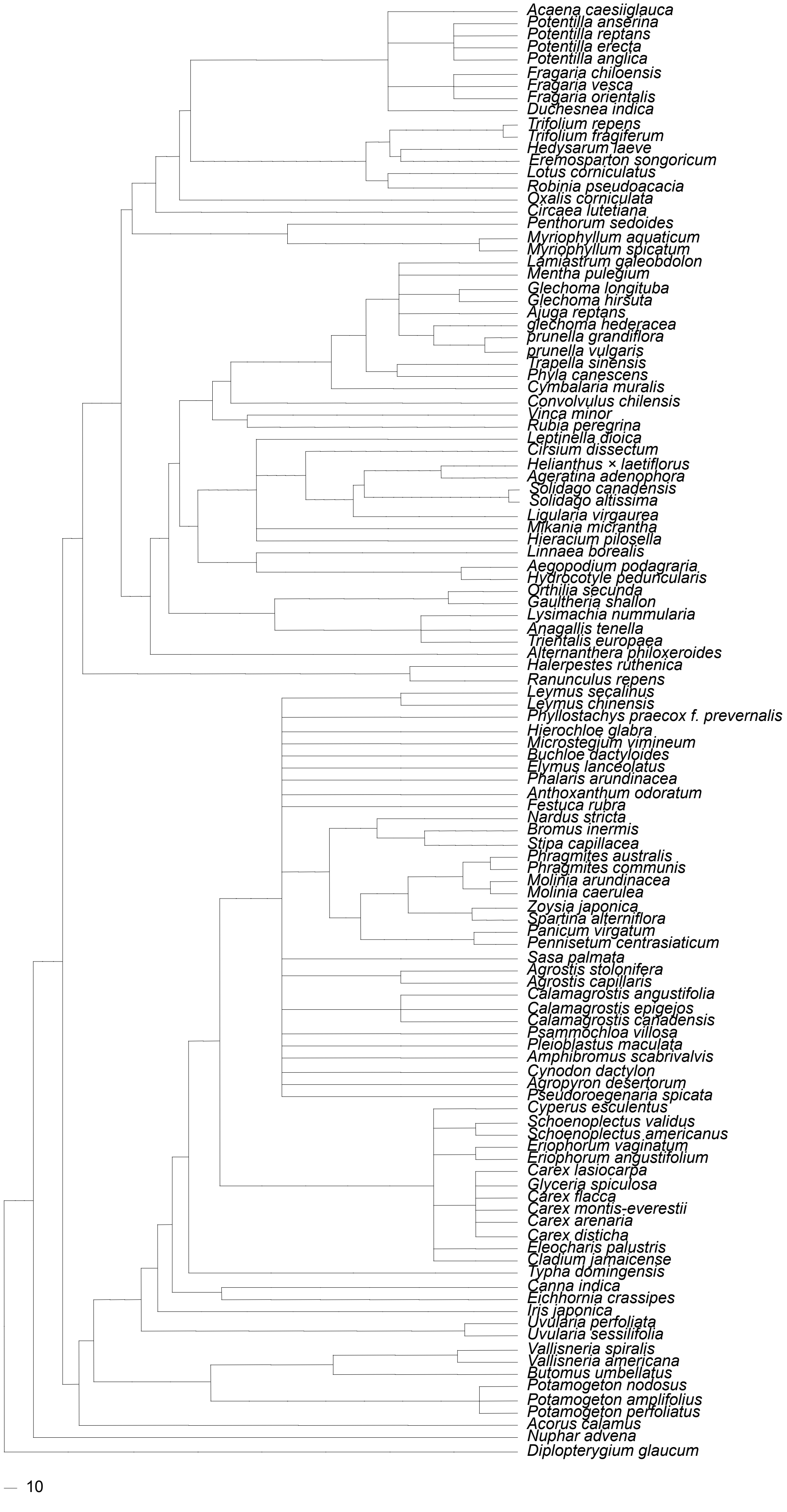

Newick:

((((((((((((((((((((((((Acaena_caesiiglauca:50.965595)Acaena:50.965595,
(Potentilla_anserina:50.965595,Potentilla_reptans:50.965595,Potentilla_erecta:50.965595,Potentilla_anglica:
50.965595)Potentilla:50.965595,
(Fragaria_chiloensis:50.965595,Fragaria_vesca:50.965595,Fragaria_orientalis:50.965595)Fragaria:50.965595,
(Duchesnea_indica:50.965595)Duchesnea:50.965595)rosaceae:50.965599)rosales:50.965591):50.965591,
((((((((((((((((((((((((Trifolium_repens:12.513873,Trifolium_fragiferum:12.513873)trifolium:12.513873):12.51387
2):12.513874):12.513874):12.513870):12.513878):12.513870,
((((((Hedysarum_laeve:15.168331)hedysarum:15.168331):15.168331):15.168331):15.168335):15.168327,
(((((((((Eremosparton_songoricum:9.100999)eremosparton:9.100999):9.100998):9.101000):9.100998):9.1009
98):9.101002):9.100998):9.100998):9.100998):9.100998):9.100998)irlc:9.101006,
((((((Lotus_corniculatus:16.901855)lotus:16.901855):16.901855):16.901855)loteae:16.901855):16.901855,
((((Robinia_pseudoacacia:20.282227)robinia:20.282227):20.282227):20.282227)robinieae:20.282227):16.901
855):9.100998):9.100998):9.100998):9.100998):9.100998):9.100998):9.100998):9.100998)papilionoideae:9.1
00998):9.100998):9.100998):9.100998)fabaceae:9.101013):9.100998)fabales:9.100998):9.100983,
(((((((Oxalis_corniculata:32.991119)Oxalis:32.991119)oxalidaceae:32.991119):32.991119):32.991119)oxalidale
s:32.991119):32.991119)celastrales_to_malpighiales:32.991119):9.101013)fabids:9.100983,
(((((((Circaea_lutetiana:35.266369)Circaea:35.266369)onagraceae:35.266365):35.266373):35.266373)myrtales
:35.266357):35.266373)malvids:35.266373):9.101013)rosids:9.100983,
((((((Penthorum_sedoides:60.066589)Penthorum:60.066589)crassulaceae:60.066589,
(((((Myriophyllum_aquaticum:30.033295,Myriophyllum_spicatum:30.033295)Myriophyllum:30.033295)halora
gaceae:30.033295):30.033295):30.033295):30.033295):30.033295):30.033295):30.033295)saxifragales:30.033
295):9.101013,((((((((((((((((((((Lamiastrum_galeobdolon:46.226414)Lamiastrum:46.226414,
(Mentha_pulegium:46.226414)Mentha:46.226414,
(Glechoma_longituba:46.226414,Glechoma_hirsuta:46.226414)Glechoma:46.226414,
(Ajuga_reptans:46.226414)Ajuga:46.226414,
((((((glechoma_hederacea:13.207547):13.207547):13.207548):13.207546):13.207546,
((((prunella_grandiflora:13.207547):13.207547,prunella_vulgaris:26.415094)prunella:13.207548):13.207546):
13.207546):13.207550):13.207542)lamiaceae:13.207550):13.207542,
((((Trapella_sinensis:23.773584)Trapella:23.773584)pedaliaceae:23.773586):23.773582,
(((Phyla_canescens:23.773584)Phyla:23.773584)verbenaceae:23.773586):23.773582):23.773582):13.207550):
13.207550,
((Cymbalaria_muralis:48.427673)Cymbalaria:48.427673)scrophulariaceae:48.427673):13.207550):13.207535):
13.207550):13.207550):13.207550)lamiales:13.207550,
((((Convolvulus_chilensis:44.905663)Convolvulus:44.905663)convolvulaceae:44.905655):44.905670)solanales:
44.905655):13.207535,
((((((Vinca_minor:35.220123)Vinca:35.220123)apocynaceae:35.220123):35.220123):35.220123):35.220123,
(((((((Rubia_peregrina:26.415092)rubia:26.415092):26.415092):26.415092):26.415100):26.415085)rubioideae
:26.415100)rubiaceae:26.415085)gentianales:26.415100):13.207550)lamiids:13.207550,
(((((((((Leptinella_dioica:101.886795)Leptinella:101.886795,
((((((((((((((((((Cirsium_dissectum:11.859838)cirsium:11.859838):11.859837):11.859840):11.859837):11.85983
7):11.859840):11.859840):11.859833):11.859840):11.859840):11.859833):11.859848):11.859833,
((((((((((((((((((Helianthus_×_laetiflorus:10.062893)helianthus:10.062893):10.062893):10.062893):10.062893):1
0.062893,
((((((Ageratina_adenophora:8.625337)ageratina:8.625337):8.625338):8.625336):8.625336):8.625340):8.62533
6):8.625336):8.625336):8.625336):8.625343):8.625336):8.625336):8.625336,
(((((((((((((((Solidago_canadensis:7.547170,Solidago_altissima:7.547170)solidago:7.547170):7.547171):7.54716
9):7.547169):7.547173):7.547169):7.547169):7.547173):7.547165):7.547173):7.547173):7.547165):7.547173):
7.547173):7.547165):7.547165,
((((Ligularia_virgaurea:25.660376)ligularia:25.660376):25.660374):25.660378):25.660378):7.547180):7.54716

5):7.547165):7.547180):7.547165):7.547165):7.547180):7.547165):7.547165):7.547180,
(Mikania_micrantha:101.886795)Mikania:101.886795,
(Hieracium_pilosella:101.886795)Hieracium:101.886795)asteraceae:7.547165):7.547165):7.547180):7.547165
):7.547165)asterales:7.547180,
((((((Linnaea_borealis:40.754719)Linnaea:40.754719)caprifoliaceae:40.754715)dipsacales:40.754723):40.7547
15,((((((((Aegopodium_podagraria:22.641510)Aegopodium:22.641510,
(Hydrocotyle_peduncularis:22.641510)Hydrocotyle:22.641510)apiaceae:22.641510):22.641510):22.641510):2
2.641510):22.641510):22.641510)apiales:22.641510):22.641510):22.641510):7.547150)campanulids:7.54718
0):7.547180,(((((((((Orthilia_secunda:27.169811)Orthilia:27.169811,
(Gaultheria_shallon:27.169811)Gaultheria:27.169811)ericaceae:27.169815):27.169807):27.169815):27.16981
5):27.169815,((((Lysimachia_nummularia:38.037739)Lysimachia:38.037739,
(Anagallis_tenella:38.037739)Anagallis:38.037739,
(Trientalis_europaea:38.037739)Trientalis:38.037739)primulaceae:38.037735):38.037743):38.037735):27.169
800):27.169815)ericales:27.169815)ericales_to_asterales:7.547150)asterids:7.547180,
((((((((Alternanthera_philoxeroides:31.865828)Alternanthera:31.865828)amaranthaceae:31.865826):31.86582
9):31.865822):31.865829):31.865829):31.865829)caryophyllales:31.865829):7.547180):7.547150):7.547180):
7.547180)core_eudicots:7.547150)trochodendrales_to_asterales:7.547180)sabiales_to_asterales:7.547180,
(((((((Halerpestes_ruthenica:42.452831)Halerpestes:42.452831,
(Ranunculus_repens:42.452831)Ranunculus:42.452831)ranunculaceae:42.452827):42.452835):42.452835):42.
452820):42.452850)ranunculales:42.452820)eudicots:7.547150)ceratophyllales_and_eudicots:7.547180,
((((((((((((((Leymus_secalinus:91.737152,Leymus_chinensis:91.737152)Leymus:91.737152,
(Phyllostachys_praecox_f._prevernalis:91.737152)Phyllostachys:91.737152,
(Hierochloe_glabra:91.737152)Hierochloe:91.737152,
(Microstegium_vimineum:91.737152)Microstegium:91.737152,
(Buchloe_dactyloides:91.737152)Buchloe:91.737152,(Elymus_lanceolatus:91.737152)Elymus:91.737152,
(Phalaris_arundinacea:91.737152)Phalaris:91.737152,
(Anthoxanthum_odoratum:91.737152)Anthoxanthum:91.737152,
(Festuca_rubra:91.737152)Festuca:91.737152,((((((((Nardus_stricta:36.694859)nardus:36.694859):36.694862,
((((((((Bromus_inermis:12.231620)bromus:12.231620):12.231619):12.231621):12.231621):12.231617,
(((((Stipa_capillacea:12.231620)stipa:12.231620):12.231619):12.231621):12.231617):12.231621):12.231621):
12.231621):12.231621):12.231621):12.231621)bep:12.231613,
((((((((Phragmites_australis:21.405334,Phragmites_communis:21.405334)phragmites:21.405334,
(Molinia_arundinacea:21.405334,Molinia_caerulea:21.405334)molinia:21.405334):21.405334):21.405334,
(((((Zoysia_japonica:18.347429)zoysia:18.347429,
((Spartina_alterniflora:12.231620)spartina:12.231620):12.231619):12.231621):12.231617):12.231621):12.231
621):12.231621):12.231621):12.231613,((((((Panicum_virgatum:17.473742)panicum:17.473742,
(Pennisetum_centrasiaticum:17.473742)pennisetum:17.473742):17.473743):17.473740):17.473740):17.4737
47):17.473740):12.231628)pacc:12.231613):12.231628):12.231613):12.231628,
(Sasa_palmata:91.737152)Sasa:91.737152,
(Agrostis_stolonifera:91.737152,Agrostis_capillaris:91.737152)Agrostis:91.737152,
(Calamagrostis_angustifolia:91.737152,Calamagrostis_epigejos:91.737152,Calamagrostis_canadensis:91.7371
52)Calamagrostis:91.737152,(Psammochloa_villosa:91.737152)Psammochloa:91.737152,
(Pleioblastus_maculata:91.737152)Pleioblastus:91.737152,
(Amphibromus_scabrivalvis:91.737152)Amphibromus:91.737152,
(Cynodon_dactylon:91.737152)Cynodon:91.737152,
(Agropyron_desertorum:91.737152)Agropyron:91.737152,
(Pseudoroegenaria_spicata:91.737152)Pseudoroegenaria:91.737152)poaceae:12.231613):12.231628):12.231
613):12.231628,((((((Cyperus_esculentus:33.200111)Cyperus:33.200111,
(Schoenoplectus_validus:33.200111,Schoenoplectus_americanus:33.200111)Schoenoplectus:33.200111,
(Eriophorum_vaginatum:33.200111,Eriophorum_angustifolium:33.200111)Eriophorum:33.200111,

(Carex_lasiocarpa:33.200111,Glyceria_spiculosa:33.200111,Carex_flacca:33.200111,Carex_montis-
everestii:33.200111,Carex_arenaria:33.200111,Carex_disticha:33.200111)Carex:33.200111,
(Eleocharis_palustris:33.200111)Eleocharis:33.200111,
(Cladium_jamaicense:33.200111)Cladium:33.200111)cyperaceae:33.200111):33.200111):33.200119):33.2001
04):33.200119):12.231613):12.231613,
(((Typha_domingensis:64.216003)Typha:64.216003)typhaceae:64.216003):64.216003)poales:12.231628,
((((((Canna_indica:38.442234)Canna:38.442234)cannaceae:38.442238):38.442230):38.442230)zingiberales:38
.442245,
(((((Eichhornia_crassipes:38.442234)Eichhornia:38.442234)pontederiaceae:38.442238):38.442230):38.442245
)commelinales:38.442230):38.442230)commelinids:12.231628,
(((((((Iris_japonica:35.165909)Iris:35.165909)iridaceae:35.165909):35.165909):35.165909):35.165909):35.165
909)asparagales:35.165909):12.231598,
((((((Uvularia_perfoliata:41.936981,Uvularia_sessilifolia:41.936981)Uvularia:41.936981)liliaceae:41.936981):4
1.936981):41.936981):41.936981)liliales:41.936981):12.231628):12.231628):12.231628,
(((((((Vallisneria_spiralis:48.036911,Vallisneria_americana:48.036911)Vallisneria:48.036911)hydrocharitaceae:
48.036911,
((Butomus_umbellatus:48.036911)Butomus:48.036911)butomaceae:48.036911):48.036911):48.036911,
(((((((Potamogeton_nodosus:30.023069,Potamogeton_amplifolius:30.023069,Potamogeton_perfoliatus:30.02
3069)Potamogeton:30.023069)potamogetonaceae:30.023067):30.023071):30.023064):30.023071):30.023071
):30.023071):30.023056):30.023071)alismatales:30.023071):12.231598,
(((Acorus_calamus:85.621338)Acorus:85.621338)acoraceae:85.621338)acorales:85.621338)monocots:12.231
628)poales_to_asterales:7.547180)magnoliales_to_asterales:7.547150)austrobaileyales_to_asterales:7.54718
0,
((((Nuphar_advena:75.471695)Nuphar:75.471695)nymphaeaceae:75.471710):75.471680)nymphaeales:75.47
1710)nymphaeales_to_asterales:7.547180)angiosperms:7.547150)seedplants:7.547170,
(((((((Diplopterygium_glaucum:50.000000)Diplopterygium:50.000000)gleicheniaceae:50.000000)gleicheniales:
50.000000):50.000000):50.000000):50.000000)monilophyte:50.000000)euphyllophyte:1.000000;
