# Supplementary figures and images for: Biomass Allocation of Stoloniferous and Rhizomatous Plant in Response to Resource Availability: A Phylogenetic Meta-Analysis
Source: Front Plant Sci. 2016 May 4;7:603. doi: 10.3389/fpls.2016.00603 (PMC4854891; doi:10.3389/fpls.2016.00603)

Supplementary Figure S4 Funnel plot

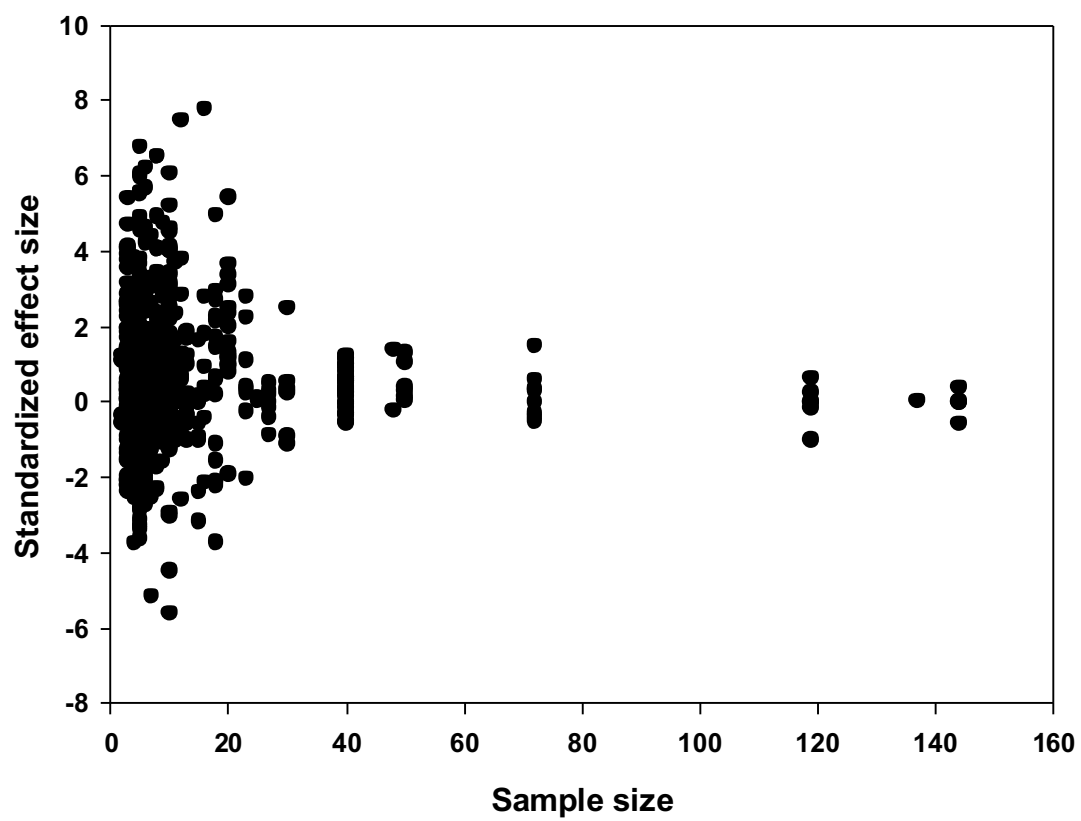

Supplement: Supplementary file 4 [file Image2.PDF]

Supplementary Figure S5 Normal quantile plot

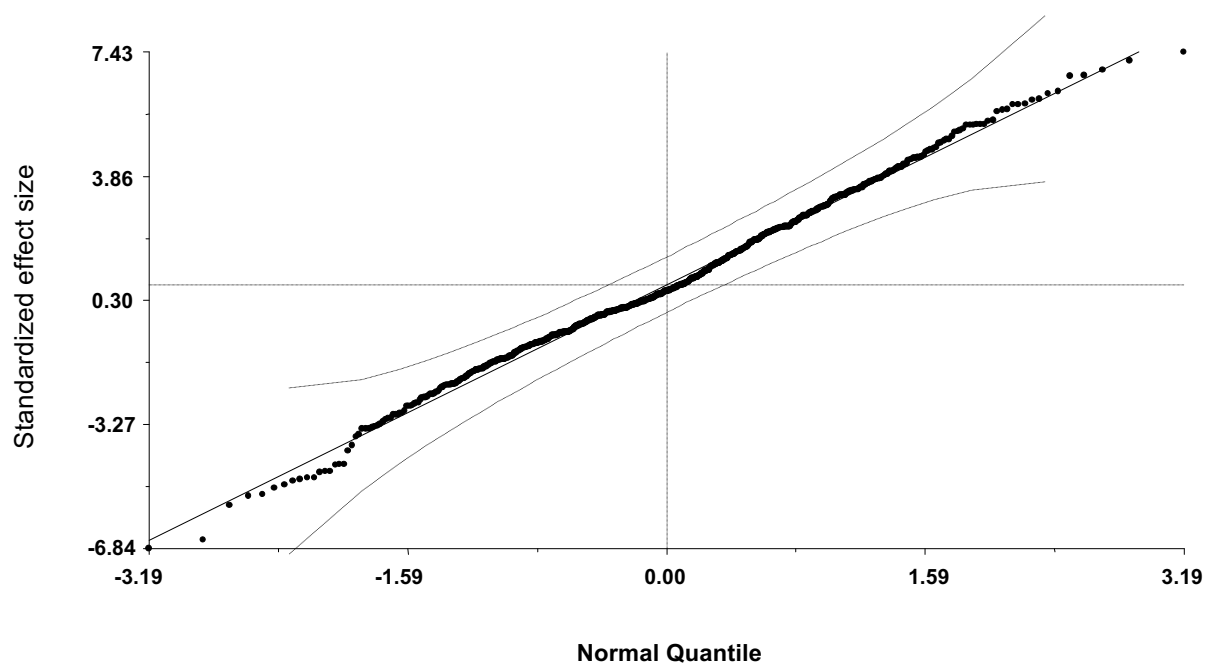

Supplement: Supplementary file 5 [file Image3.PDF]
